# Cataphoresis

**Published:** 1897-09

**Authors:** Louis Jack


THE
### International Dental Journal.

Vol. XVIII. September, 1897. No. 9.

### Original Communications.[1]

### CATAPHORESIS.[2]

BY LOUIS JACK, D.D.S.

Gentlemen,—The subject selected for your instruction this evening is the new and most interesting method of introducing medicaments into the tissues. In its application in certain directions it is proving of great importance to dentistry.

Before entering into the direct relations of the subject, it will be of interest, and throw light upon the application of this mode of treatment, if some consideration of the general principles and laws governing the peculiar force called electricity be stated. To make use of electricity for any practical purpose necessitates a definite knowledge of these principles.

The exact nature of electricity, as you are aware, has not been determined. It can only at present be defined as a kind of cosmic force; but certain properties and modes of action have been clearly determined, and have been reduced to mathematical precision. From these, definite laws have been clearly established.

The minds of most persons have been confused by the term fluid as applied to electricity, and, in connection with the word

[1] The editor and publishers are not responsible for the views of authors of papers published in this department, nor for any claim to novelty, or otherwise, that may be made by them. No papers will be received for this department that have appeared in any other journal published in the country.

[2] Lecture delivered before the E. T. Darby and James Truman Societies,—students of the University of Pennsylvania.

*current*, as expressive, by analogy, of the movement of fluids. As electricity is capable of being converted into light, heat, and magnetism, and through magnetism into mechanical force, it follows, hence, that electricity is neither matter nor a condition of matter, but is a form of force. This is further established by the principle that either of those other mentioned forces may be converted into electricity or be made the means of exciting electrical action.

As the ether is assumed to be a subtile substance, filling the immensity of space and permeating all forms of matter, in like manner electricity is considered to be an all-pervading force, equalizing the movements of and controlling all molecular action. The presence of electricity is elicited by every chemical activity and every mechanical action; each whispering breath of air excites it; the molecules of water, as they are raised by the sun's heat, bear with them their modicum of this force until the potentiality of the storm-cloud manifests the tremendous force so accumulated by the lightning's flash and the thunder of the heavens.

Its movements are constantly in the direction from bodies of high to those of low potentiality. This action, since the presence of the force is everywhere, is incessant, but is ordinarily unnoticed, except where potentials are excited by chemical or mechanical means.

Electricity has two elemental properties. These are defined as current strength, which is designated by the term ampèrage, and electro-motive force, which is termed its voltage.

*The active energy of electricity depends upon the first property,—its ampèrage, its distribution upon the latter. Since it must be assumed that few bodies are perfect conductors, this force or pressure is of that degree which is required in any given case to move the active element, the ampèrage, against the resistance it meets with.*

For illustration: the voltage has its analogue in the height of a column of water, the ampèrage being represented by an opening at the bottom of the column; according to the height of the column constantly maintained will be the force represented by the escaping stream at the bottom. The resistance would here be represented by the length of pipe which may be applied to the aperture,—in other words, by the extent of the friction.

In *perfectly conducting* substances electricity moves with absolute freedom, under any electro-motive force, however small.

In *perfectly non-conducting* substances electricity will not move under any electro-motive force, however great.

In *imperfectly conducting* substances electricity moves only under the exhibition of intense electro-motive force. The required force varies as the substance is a more or less indifferent conductor.

An ampère of current is so much as will deposit .00118 gramme of silver per second when passing through a standard solution of nitrate of silver, or which will decompose .09326 milligramme of water in one second.

Hence, the ampère is the active cause of electrical power, and depends upon the voltage for its efficiency.

A *volt* is the electro-motive force required to impel one ampère of current through one ohm of resistance.

An *ohm* is the amount of resistance which will permit one ampère of current at one volt to pass in one second.

In the economic application of electricity, its transmission is effected through metal connections, the resistance of which is governed by the character of the metal, the cross-section, and the distance. For certain purposes other substances, such as water, carbon, or graphite, are employed to effect great resistances.

The *ampèrage* flowing in a circuit is equal to the E. M. F. divided by the resistance.

The *resistance* equals the E. M. F. divided by the ampères.

The *volts* equal the ampères multiplied by the ohms.

The *watts* equal the volts multiplied by the ampères. The watt is the unit of efficiency, or of work. (Seven hundred and forty-six make one horse-power.)

The formula is expressed thus: $V \div R = A$; $V \div A = R$; $A \times R = V$; $V \times A = W$.

It follows, from the formula, that the amount of power and the cost of producing it are the same, whether the current is of large ampèrage at low voltage, or of small ampèrage at high voltage. Thus, an incandescent lamp may be supplied by one hundred volts at one-half ampère, or by fifty volts at one ampère, the result in each case being fifty watts.

Comparative Illustration.—It is the same as with water in a column; the same degree of force is secured when the column is heightened and the aperture correspondingly reduced.

To illustrate the application of the formula in practice:

If we have a current of one hundred volts at fifteen ampères, and wish to use only two and a half ampères, the resistance put in the circuit is found thus: $100\ V \div 2\frac{1}{2}\ A = 40$ ohms.

In case we have seventeen and a half volts at ten ampères, and wish to charge a storage-battery at two and a half ampères, the resistance is determined as before, thus: $17\frac{1}{2} \div 2\frac{1}{2} = 7$ ohms.

Now, if we have two and a half ampères under seven ohms resistance, you see it requires seventeen and a half volts to move this degree of ampèrage against the given resistance, thus: $2\frac{1}{2}\ A \times 7\ R = 17\frac{1}{2}\ V$.

If one has a current of one hundred and ten volts, and wishes to employ a motor of one-quarter horse-power, what is the least ampèrage required? Answer: $1\frac{69}{100}$; which is found by dividing 186 watts by 110 —.

With the formula any one may, from one known quantity and a desired quantity, determine with ease the third factor.

These elementary examples are shown you to make it plain that the means of determining the character of the current for any given purpose is very simple.

These examples make it appear that the elements of electricity are more easily calculated than are those of heat. We are lighted by the sun's rays, and are heated by derivatives from the sun, and are familiar with the employment of heat and light; but, as electricity cannot so easily be borrowed at will, it has been treated as incomprehensible by the ordinary person, but you see its elements are of simple computation.

Electrical force of different quality may be produced with galvanic batteries by arranging them in series or multiple. If in series, the voltage is the sum of the volts of the cells so arranged; the ampèrage is that of one of the cells. This arrangement produces a current of high tension.

If joined in multiple,—that is, positive to positive and negative to negative,—the volume in ampères is the sum of the ampères of all the cells, while the voltage is that of one of the cells. This method produces a low tension current.

When arranged in series, if each cell has a voltage of two and an ampèrage of one, the current with five cells will have an electromotive force,—*i.e.*, voltage of ten, and a strength of one ampère. This method is called that of intensity, as just stated. The same number of cells joined in multiple would yield a current of five ampères or two volts, and hence would be of low tension, but of high quantity.

In cataphoresis, as applied in dentistry, where batteries are the source of the current, the arrangement should be in series, since, as will later appear, the teeth are tolerant of the movement into them of only a small degree of ampèrage. The ampèrage must necessarily be of low ratio to the voltage.

We now approach the practical features of the application of electricity to the treatment of tissue by conveying thereby drugs into their substance.

Electricity in connection with medicaments has in dentistry several directions in which it may be employed,—viz., for the treatment of acute dentinal sensitivity, for anæsthetizing and removal of the dental pulp, for relieving deep inflammatory conditions of the tissues adjacent to the teeth, for bleaching discolored teeth, and for disinfecting root-canals.

Your attention will be principally directed to the treatment of dental sensibility.

The term "voltaic narcotism" was the first term applied to the phenomena observed; then, cataphoresis; later, the term electrical osmosis, and other terms, as, anaphoresis, electro-medicamental diffusion.

The term electrical osmosis in most respects better defines the action which takes place than the word cataphoresis.

You are already familiar with the phenomena of endosmosis. Briefly stated, it is that when two fluids of unequal density are placed near each other with a permeable division between them, such as a membrane, the two substances become mixed through the partition. If a solution of a salt is on one side and water on the other side, the water at first passes towards the solution and raises its volume, but at length the two become equalized of strength.

If we arrange a vessel containing a saline mixture on one side and water on the other, and apply the anode on the side of the salt and the cathode on the side of the water, the osmotic process is prevented. If, on the other hand, the anode is placed on the side of the water and the cathode in the salt, the osmotic action is greatly accelerated, and what otherwise would require hours to naturally effect is done immediately.

This shows that electricity has the power to overcome or accelerate the natural law of osmosis.

But to make the matter clearer to you and to still better explain the phenomenon of the conveyance of a drug into the tissues: If a substance containing water, as a cooked egg, a ball of wet clay, or a piece of muscular tissue, has an anode connected with a current of high potential attached to one side, and the cathode is attached to the other side, the watery contents of the substance is conveyed forward and appears in excess at the side on which the cathode is placed; at the same time the anodal surface becomes dried, also, if a capillary tube of glass is filled with water having an anode applied in one end and a cathode at the other end the water will flow against gravity. As a membrane or a tissue may be considered to be a series of tubes in close contiguity, it is at once apparent to you that the movement of fluids must take place through them in the direction the current is passing.

These experiments demonstrate the action which takes place when an aqueous solution of cocaine, for instance, is placed in a carious cavity. As the anode is put in contact with the saturated cotton the fluids of the tooth advance towards the pulp, through the canaliculi, their place being taken by the solution of cocaine. At the same time it is observed the saturation of the pledget of lint becomes less wet and requires an occasional addition of the solution to the lint. Some loss of the water in the solution is to be accounted for by the evolution of heat in the parts immediately concerned, because of their resistance to the current.

We have then two coincident phenomena to keep in view: the movement of the fluids of the dentine along the course of the current, and the well-observed law of the movement of any fluids in the range of the current from positive to negative.

You thus have presented to you another law of electricity, which has not been sufficiently emphasized as a basis for a correct hypothesis to account for the transmission of an anæsthetizing agent.

As we have entered the practical field of the subject, I will take up the matter in the sequence that appears in its clinical aspects.

As to the character of cases where electrical osmosis is required, it is only necessary to state that it is applicable to those of acute condition,—that is, in a state of hyperæsthesia. It would be questionable to go through the required procedures where an ordinary escharotic would suffice, or where the application of heated air would render the case easily treated; but in hypersensitive cases electrical osmosis is the most benign benefit which has been conferred upon dentistry in the century, equalling in its field with us the glorious and humane results which attend the use of general anæsthesia in major surgery.

When a case presents requiring this treatment, the tooth should be isolated by means of rubber dam. Should the position be on a proximate surface, the neighboring tooth should be included, unless the adjacent surface contains a metallic filling.

To illustrate the importance of this, let me state that a few days since I unwittingly passed the rubber dam over two teeth where one had a gold filling, recently put in. The gold filling was varnished. The cocaine applied and current turned on from ten cells at an indicated ampèrage of three-twentieths milli, for thirteen minutes, with no result. There was evident leakage through the gold filling from imperfect insulation. A new dam was applied, enclosing only the carious tooth. The current again being applied for ten minutes, there was complete relief of the sensitivity.

In applying the dam the tooth should be ligated with a well-waxed thread, and any other means which may suggest themselves be used in difficult cases to prevent the encroachment of moisture.

The anæsthetizing agent may be either cocaine hydrochlorate or cocaine citrate, of a strength of from twelve to twenty-four per cent. in distilled water. The first named should be made fresh each day or time. The most convenient method is to have the cocaine prepared in powders of one and one-fifth grains. If to this quantity be added five minims of water, the result will be twenty-four per cent., and with ten minims, twelve per cent.,—the druggist's minim graduated glass being a most convenient vessel in which to make the combination.

The applicator, which forms the positive pole and is otherwise called the anodal electrode, should have a point of iridiumized platinum, for the reason that platinum is not oxidizable, and is not acted upon by hydrochloric acid. The iridium confers the necessary stiffness. The size of the point should be as broad as permissible to easily enter the cavity and to cover as much of the lint containing the cocaine as may be convenient.

An excellent form of anode is to curl a piece of fine platinum wire into a flat knot, or to form it in a double loop. On the loop sufficient lint to fill the cavity is wrapped, when it may be forced into those on the occlusal and proximate surfaces. These simple applicators are connected with the usual cords by a spring clip furnished by Louis Costa & Co., of this city. The latter method is used to enable the cord conveying the current to be sustained in order to avoid holding the applicator with the hand. This is for the sake of avoiding fatigue, and also, more important than this, to secure perfect stillness of the anodal connection, since, if any movement takes place, some shock is liable to be sustained.

Various forms of the anodal end of the applicator will suggest themselves to any one.

The cathodal electrode (negative) should be placed at some indifferent place, regard being had to lessen the resistance as much as may be possible. The resistance on the cathodal surface is of the skin and adipose tissue. If the person be comparatively lean, the cheek before the ears is an excellent position; if fat, the palm of the hand offers probably less resistance, as being less fat.

SIZE.—If placed on the cheek, it should be not less than one and one-half inches in diameter; if in the hand, the ordinary hand cathode is used, covered with a napkin. In each case the spunk or the napkin should be wet with a solution of sodium chloride.

We now reach the question of the greatest importance,—the character, the voltage, and the ampèrage of the current.

It is with reference to clear views on this point that I felt it necessary to outline the general principles concerning electricity. It is easy to cause pain in the exhibition of the current if care be not taken, and it is not difficult to conduct the treatment without distress, and to produce results eminently satisfactory in nearly every case.

The first requisite is that the current be direct and constant. If sudden fluctuations, however small, occur, either of the voltage or of the ampèrage, immediate shock is felt in proportion to the range of the variation.

The source of current frequently used has been the one-hundred-and-ten-volt direct Edison current. This current, as will later appear, is of unnecessarily high voltage; is subject to some changes of voltage and to constant variations of ampèrage, as load is taken off or put on the general circuit. The variations of voltage are small, because the effort at the central stations is to maintain the electro-motive force at an even rate. This is necessary, as outlined in the beginning, because the efficiency of the system depends upon the maintenance of the pressure. But the ampèrage is never constant, and, as shown you in the premises, it is this property which is the prime element of electrical force. It is more important to avoid fluctuations of ampèrage than changes of voltage.

There are other objections to the one-hundred-and-ten-volt current, connected with possible grounding through water-pipes, through possible crossing of wires, etc.

Besides, you will probably agree with the conclusions, which we will come to presently, that the one-hundred-and-ten-volt current is employing a club to do what a wand will more efficiently effect.

The opinion is more and more becoming a fixed one that the current supplied by the public electrical companies is unreliable and unsafe.

For electrical osmosis the better source is from some form of galvanic cell arranged in series,—that is, positive with negative.

The amount of voltage required is from five to twenty. Less than five appears to be insufficient, and more than twenty are rarely needed. The ampèrage required for the series is not over one-half ampère, which will appear later when it is shown how infinitesimal a rate of ampèrage is sufficient and is tolerable.

The degree of electrical energy tolerated by living dentine is exceedingly small, on account of the peculiar and intense pain excited by the transmission of slight electrical currents through the teeth. This is shown by the low initial voltage required and the small degree of ampèrage used in cataphoric apparatus. Even these are generally greatly in excess, and have to be reduced by methods of effecting resistance.

The effect of resistance is to diminish the degree of ampèrage to a much greater extent than the voltage. I have made the endeavor to show that it is the ampèrage which produces the effect, and shall endeavor to make clear that it is the ampèrage which excites in the tissue the sense of pain induced by the evolution of heat. A comparative high rate of voltage will be tolerated when the degree of ampèrage is extremely small. Tesla has stated that he has produced in the vacuum-tube a voltage of one million volts, which he has passed through his body without injury. This current necessarily had an infinitesimal ampèrage, which rendered such an experiment possible.

In electrical osmosis as applied to the teeth the resistance necessarily must be extremely great to prevent what is called the impulse of the current, and also to diminish the ampèrage to a tolerable degree, as well as to restrict the voltage,—in other words, to diminish the energy of the current to sufficient weakness to meet the requirements of the most impressible cases which occur.

All forms of resisters are constructed on the principle of the selection of materials which are very imperfect conductors of electricity. The available substances for this appendage to a cataphoric apparatus are water, carbon, graphite, and coils of wire of known high resistance, the metal offering these requirements being German silver. In the case of the latter the degree of resistance is regulated by the length and fineness of the wire. In comparison with silver, the best conductor, German silver has a resistance nearly fourteen times as great (13.92).

The water rheostat may be passed as inconvenient, because the column required must be extremely high. German silver coils may also be refused as adjuncts, except when it may be necessary to reduce the ampèrage and voltage of high currents before connecting them with any special cataphoric apparatus. It may, however, be used in connection with a *shunt*, as described by Dr. Price in the February number of the *Dental Cosmos*, to which you are referred.

The carbon and graphite resisters are usually connected in the form of a ring, with a switch passing over the circular path, or by arranging a series of points in close proximity, each point being in contact with the ring of graphite. In these forms they are called controllers. This is probably the most available form of resistance for our purposes. The latter form is the one I have been using, as invented by Willms; this has a rheostat of one hundred and twelve points, with which the switch engages as it passes over the circle. The one in the instrument before you has a resistance of four hundred thousand ohms, which is greater than is actually required. A German silver resistance of four hundred thousand ohms capacity would fill a very large portion of this room. For our purposes the initial ampèrage need not be over one-quarter of an ampère, which the present controller is sufficient to reduce without any apparent heat being excited in the instrument. This method of construction here shown you gives a fine gradation of resistance.

An indispensable instrument is the milammeter, which should have a scale indicating degrees of ampèrage as low as one-twentieth of a milliampère (a milliampère is one thousandth of an ampère).

In the arrangement of the apparatus to effect electric osmosis, the battery, the controller, the milammeter, and the patient are arranged in series.

In the analysis of the course of the current it is seen that the dentine is also a resister, and a most remarkable one at that, the resistance of the dentine varying, according to Dr. Price, from twenty to seventy thousand ohms, the differences being due to the distance of the pulp and to density of structure.

The result of the resistance of the dentine, unless the voltage is low and the ampèrage infinitesimal, is that at the closing of the circuit a slight shock is felt in some cases and the current may maintain some continued pain. The sense of pain occurs with different individuals at different rates of electro-motive force and at varying degrees of ampèrage.

The first sensation when the current has too much pressure is shock, and appears to be due to what is called impulse of the current. You will readily perceive from the law stated that electricity will not move through bodies which are non-conductors, under any degree of electro-motive force however great; but you have daily observation that in the arc lamp the current leaps through the space between the carbons. This is due to impulse.

That the shock is due to impulse is shown in that in a moment, in a twinkling, this ceases and more current may be permitted to pass without pain until a definite degree of ampèrage becomes apparent.

When this latter sign of electrical irritation appears we have to consider, from the laws of electricity, that it is due to the evolution of heat, caused by resistance to the current.

There is great variation in persons as to the degree of ampèrage recorded when this form of irritation appears. With some it is at one-tenth milliampère; with others, it is not noticed until four-tenths are observed.

How shall we account for this difference where there probably is not a great variation in the tissue resistance? There is so little difference in the density of dentine that we have to seek for some other cause than difference of tooth resistance. You are aware of the well-recognized susceptibility of the teeth of different individuals to thermal shock. This sensibility of the teeth to elevation of temperature or to its reduction has been called the temperature sense of the teeth, and is a very variable state.

Some patients compare the electrical irritation to cold, notwithstanding the sensation must be caused by increased heat. This is to be accounted for by the fact that the usual form of thermal irritation is caused by reduction of temperature; as they have no comparison of the effects of heat, and not having the tongue or contiguous parts to guide them as to the temperature, they attribute it to the sensation with which they are familiar.

In the application of current, the occurrence of the first trace of electrical irritation—heat-pain, in other words—is designated as the pain limit. This should not be passed until there are indications, by the experiment, of very slightly reducing the resistance, that more current may be added. If this increases the irritation, the controller should he turned back a degree.

I think I have sufficiently indicated to you that the pain limit is due to the degree of ampèrage, and that this occurs with different persons at varying degrees of the ampèrage, and to the same person at a certain ampèrage regardless of the rate of the voltage.

You may here ask, What are the indications when the current has been continued with sufficient duration to effect the purpose of the administration? Do you wait until the electrical irritation ceases? In some instances, where there is little electrical irritation, where, indeed, twenty volts will be tolerated, and where the record of the ampèrage is four-tenths of the milliampère, one may after eight or ten minutes carry the switch of the controller nearly the full circuit without disturbance; but, in general, when the pain limit occurs at one-tenth milli this result cannot be expected. In these cases the only measure is that of time, all the while keeping on the border of the pain limit. The usual time required is from ten to thirteen minutes, although I have known complete anæsthesia to occur after six minutes. In general terms, where the tolerated degree of ampèrage is low, more time is required than where three- to four-tenths milli is admissible.

In respect of electrical irritation, some value must be placed upon the high nervous sensibilities of some persons, as with these there usually appear greater susceptibility to electrical irritation.

The following table of calculated resistances makes it appear how considerable is the liability to the generation of heat in the dental tissues, in view of their density, and commands caution in the application of electrical force for the purpose in view:

| | |
|---|---|
| With 15 volts initial pressure at $\frac{4}{10}$ milli, the ohms are | 37,500 |
| With 15 volts initial pressure at $\frac{1}{10}$ milli, the ohms are | 150,000 |
| With 10 volts initial pressure at $\frac{4}{10}$ milli, the ohms are | 25,000 |
| With 10 volts initial pressure at $\frac{1}{10}$ milli, the ohms are | 100,000 |
| With 5 volts initial pressure at $\frac{4}{10}$ milli, the ohms are | 12,500 |
| With 5 volts initial pressure at $\frac{1}{10}$ milli, the ohms are | 50,000 |

As the varying resistance of the body, including the dentine, varies from twenty thousand ohms to seventy thousand, it appears from these calculations to be necessary that the controller should have at the highest point a resistance of at least one hundred and fifty thousand ohms to avoid the impulse of the current at the moment of closing the circuit. My experience, as before indicated, is that one hundred thousand ohms is not nearly sufficient.

The varying resistance through the body depends upon the density of the dentine, the distance the current traverses, the condition of the surface of the skin, as to its cleanness, its dryness, and the thickness of the adipose tissue at the surface where the cathode is placed.

The average resistance of the body, according to Dr. Price, is over twenty-five thousand ohms from cavity to hand. The difference of resistance from surface of tooth to hand and to the cheek is from three thousand to five thousand ohms. He reports a case where the resistance from cavity to hand, with a fifty-per-cent.

solution of cocaine, was twenty-eight thousand five hundred ohms, when, on placing the pad on the cheek, it was reduced to twenty-three thousand ohms. Hence the cheek is preferable to the hand.

But we must consider here the depth of the fatness of the cheek, and determine whether the palm, on account of the absence of fat there, may not give less resistance by the longer distance in the condition of deep adipose on the cheek.

Dr. Price further places the average resistance from hand to tongue at nine thousand ohms, and from cheek to tongue at three thousand ohms. This determines the average resistance of dentine to be about twenty-five thousand ohms.

The components of the anæsthetizing agent, as well as the percentage of the solution of the medicament, have a qualifying influence, as appears from the experiments of Dr. Price, where a section of dentine partially dry had a resistance of thirty thousand ohms; that, after being dried and saturated with a forty-per-cent. solution of cocaine, the resistance was reduced to four thousand five hundred ohms. This partially accounts for the slight increase of the degree of ampèrage after the cocaine has been continued for some time without increase of voltage.

The principles I have endeavored to make clear to you, and the facts presented in connection with them, demonstrate the importance of careful selection of the rate of initial voltage to be applied, and the use of a current of relatively low ratio of ampèrage to the voltage by the intervention of a controller of very high resistance; of the necessity to control the current a little within the boundary of the pain limit; of the avoidance of impulses of current by accidental displacements or movements of the anodal electrode, and of the maintenance of a constantly moist state of the medicated lint.

I will now come to a conclusion by directing your attention to some of the practical details of the administration.

The arrangement of the apparatus is to have the battery, the controller, the milammeter, and the patient in series,—the anode (positive) being connected with the cavity by the applicator, the cathodal (negative) conductor on the cheek or hand. The current is then turned on, selecting a voltage of ten to fifteen. The controller being at zero, the switch is gradually advanced until the approach of the pain limit is apparent. When this is at a low degree of ampèrage, say one-tenth milli, one should advance cautiously and wait until more strength of current will be tolerated, as nothing is gained by forcing beyond the condition of easy tolerance, since, generally, the desired result is reached with the same satisfaction as in cases where, at the commencement of the pain limit, the milli may be three- to four-tenths. To those commencing the use of this method of treatment, cautious action is necessary, and, above all things, such persons should have studied well the general principles involved.

An apparent ampèrage of five-tenths milli is to be regarded as one of question as to the isolation of the tooth, and indicates leakage at the cervix. If it should be six-tenths, this becomes a certainty. If so, when this cannot be remedied, the proper course is to increase the voltage and cautiously continue the administration for a longer time than the usual period.

When the switch of the controller, after, say, six to eight minutes, may be advanced several points without transgressing the pain limit, it is positive indication, when no leakage exists, that the dentine has become anæsthetized, but it does not follow that relief does not come without this advancement, as cases appear where no change in the controller can be made, and where the recorded ampèrage is only one-tenth milli, that absolute relief is found after a continuance of ten to fifteen minutes of treatment.

When the indications point to conclusion, the switch should be slowly carried back to zero, and on removal of the connections the excavation may be made.

In some cases, while the major portion of the preparation of the cavity may be effected without pain, we may be denied the cutting of deep grooves at the margins, and may have to make a second application. This, however, is rarely necessary. If so, a short time only is required.

In this connection appears an interesting consideration. The question may arise in your minds, Why is it that, as electricity moves along the lines of least resistance, it does not take the direct course towards the pulp, through the dentine at the pulp-floor of the cavity, and avoid the portion of the dentine at the margins? But experience shows that this is not the case. The current is evidently diffused, and acts upon the margins, and universally does so. It evidently seeks the channels of the canaliculi in the stratum granulosum. It has been claimed by some that it reaches this portion of the dentine by first approaching the pulp, and, by anæsthetizing this organ, permits excavation to be made near the stratum granulosum. If this were the case, all parts of the margins, however deep and at whatever part of the cavity, would be acted upon, and would be insensitive. This is disproved by the fact that in some cases, by deep grooving at the margins, we arrive at a point beyond the sphere of the anæsthesia. In addition, the point where we most frequently find the recurrence of sensibility is at the occlusal margin, which is the one where, by the general direction of the current, we might expect some sensibility. If the pulp were first acted upon, there should be no recurrence here.

This brings up another question. Shall we remove the caries from the cavity before commencing the treatment? On this point I have not reached definite conclusions. It has agreed, however, with my general observation, that it is better not to do so,—at least no further than to determine whether the sensibility is of such a degree as to necessitate the use of cataphoresis.

I have gone over this interesting subject in all its general features, and with particularity in those where this has appeared useful to beginners. There is so much that is of abounding importance connected with it that it is hard to find the point where there is not matter of interest and profit to state. No subject since the beginning of dentistry has caused so much discussion in so short a time, or which has excited such universal attention.

Therefore, it is my trust that I have not discussed it without a responsive interest on your part.

